# Density Functional Theory Unveils the Secrets of SiAuF_3_ and SiCuF_3_: Exploring Their Striking Structural, Electronic, Elastic, and Optical Properties

**DOI:** 10.3390/molecules29050961

**Published:** 2024-02-22

**Authors:** Fekhra Hedhili, Hukam Khan, Furqan Ullah, Mohammad Sohail, Rajwali Khan, Omar H. Alsalmi, Hussein Alrobei, Khamael M. Abualnaja, Ghaida Alosaimi, Hissah Saedoon Albaqawi

**Affiliations:** 1 Department of Physics, College of Science, University of Ha’il, P.O. Box 2440, Ha’il 81451, Saudi Arabia; f.hedhili@uoh.edu.sa (F.H.); h.albuqawi@uoh.edu.sa (H.S.A.); 2 Department of Physics, Faculty of Science, Al Manar University, 1060 Tunis, Tunisia; 3 Department of Physics, University of Lakki Marwat, Lakki Marwat 28420, Khyber Pakhtunkhwa, Pakistan; 4 Physics Department, Faculty of Applied Science, Umm AL-Qura University, Makkah 24382, Saudi Arabia; 5 Department of Mechanical Engineering, College of Engineering, Prince Sattam bin Abdul Aziz University, Al-Kharj 11942, Saudi Arabia; 6 Department of Chemistry, College of Science, Taif University, Taif 21944, Saudi Arabia; k.ala@tu.edu.sa (K.M.A.); g.alosaimi@tu.edu.sa (G.A.)

**Keywords:** fluoride perovskite, density functional theory, electronic properties, structural properties, optic behaviors

## Abstract

In the quest for advanced materials with diverse applications in optoelectronics and energy storage, we delve into the fascinating world of halide perovskites, focusing on SiAuF_3_ and SiCuF_3_. Employing density functional theory (DFT) as our guiding light, we conduct a comprehensive comparative study of these two compounds, unearthing their unique structural, electronic, elastic, and optical attributes. Structurally, SiAuF_3_ and SiCuF_3_ reveal their cubic nature, with SiCuF_3_ demonstrating superior stability and a higher bulk modulus. Electronic investigations shed light on their metallic behavior, with Fermi energy levels marking the boundary between valence and conduction bands. The band structures and density of states provide deeper insights into the contributions of electronic states in both compounds. Elastic properties unveil the mechanical stability of these materials, with SiCuF_3_ exhibiting increased anisotropy compared to SiAuF_3_. Our analysis of optical properties unravels distinct characteristics. SiCuF_3_ boasts a higher refractive index at lower energies, indicating enhanced transparency in specific ranges, while SiAuF_3_ exhibits heightened reflectivity in select energy intervals. Further, both compounds exhibit remarkable absorption coefficients, showcasing their ability to absorb light at defined energy thresholds. The energy loss function (ELF) analysis uncovers differential absorption behavior, with SiAuF_3_ absorbing maximum energy at 6.9 eV and SiCuF_3_ at 7.2 eV. Our study not only enriches the fundamental understanding of SiAuF_3_ and SiCuF_3_ but also illuminates their potential in optoelectronic applications. These findings open doors to innovative technologies harnessing the distinctive qualities of these halide perovskite materials. As researchers seek materials that push the boundaries of optoelectronics and energy storage, SiAuF_3_ and SiCuF_3_ stand out as promising candidates, ready to shape the future of these fields.

## 1. Introduction

A basic component for the survival of mankind in this world is energy, which is always available in specific average quantities to everyone. Today, for generating and storing energy, crystals called perovskites are used, because of which this field is always very attractive to researchers. So, for obtaining perovskites with valuable results, they are introducing new perovskites. The substance composition of perovskites is denoted as ABX_3_, where X has the potential to be replaced by elements such as oxygen, chlorine, bromine, fluorine, or iodine. The initial two components in the formula are cations A and B, while the last component, X, serves as an anion. The inaugural perovskite material discovered, CaTiO_3_, adheres to this specified formula. In the structural framework of each perovskite, an anion is intricately linked to 12 cations labeled as “A” and 6 cations labeled as “B”. Oxide perovskites can manifest as insulators, conductors, or semiconductors. These compounds exhibit a stable crystalline structure and boast commendable electronic properties. Research has revealed their applicability in diverse fields, including the lens industry, lithography, and the development of photoelectric resources and the creation of energy storage devices. Notably, these materials have demonstrated high efficiency in these applications [1,2,3]. Numerous studies have been performed to examine the range of distinctiveness of perovskite crystals, particularly oxide perovskites, which are the primary focus of interest. According to research, these substances are elastically anisotropic and mechanically stable [4,5,6]. The most important uses of ABF_3_ crystals include photovoltaic components and for electronics, automobiles, and optics as the best storage medium of energy, and this application indicates efficient and stable hydrogen production [7,8,9]. For oxide perovskites to be stable, with inorganic or organic elements or metals from transition metals, oxygen is put together. Broad band-gap oxide perovskites are the best options. In the making of glass, which can work in the wavelengths of vacuum ultraviolet (VUV) and ultraviolet (UV) [10,11], such molecules can be used because of their good band gap and high potential. Nowadays, halide perovskites are the main interesting materials for researchers [12,13,14,15,16,17,18,19,20,21,22]. Harmel et al. [15], to study the behaviors of barium-based BaCsF_3_ oxide perovskites using DFT, conclude that BaCsF_3_, due to their enormous direct band gap and insulating behavior in the ultra violet range, as well as ensembles of their unreal component, will be good for opto-electronic devices. Daniel et al.’s [16] review of a few characteristics of LiBaF_3_ showed that such materials are good for storing energy.

We have found that materials having band gaps greater than 3.1 eV will perform well in the UV spectrum [22]. SiAuF_3_ and SiCuF_3_ look like metallic compounds. Here, using DFT and the FP-LAPW method, we will study the basic properties of SiJF_3_ (J = Au and Cu) halide perovskites, so that it may be helpful for further research work. These properties are structural, elastic, electronic, and optical.

## 2. Results and Discussion

Here, our Tb-Mbj potential procedures’ output is extensively scientifically examined. We will talk about structural and optical aspects using this methodology.

### 2.1. Structural Properties

For our study, we chose SiJF_3_ (J = Au and Cu). Pm-3m number 221 is the space group of our selected crystals. Its unit cell structure is cubic, which consists of a single molecule, and the following state could fit.

**Figure 1:**
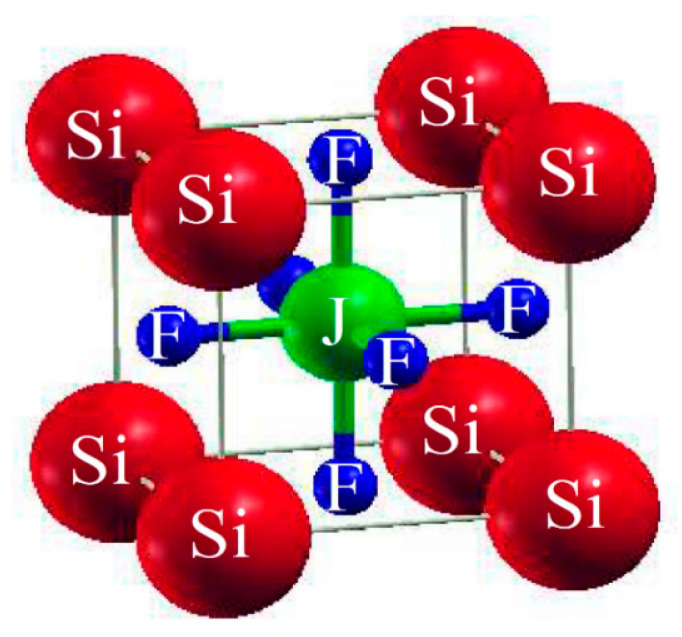
Typical cubic crystal structure of ternary molecules SiJF_3_ (M = Au, Cu).

The ABF_3_ formula shows that they are fluoride perovskites. The atoms within the unit cell are arranged with silicon (Si) atoms positioned at coordinates (0, 0, 0), gold (Au) and copper (Cu) atoms (denoted as J atoms) occupying positions (1/2, 1/2, 1/2), and fluorine (F) atoms located at (0, 1/2, 1/2), (1/2, 0, 1/2), and (1/2, 1/2, 0), as illustrated in Figure 1. The comprehensive energy around the equilibrium volume (V_o_) is to be determined in comparison of the unit-cell volume. To facilitate volume optimization for predicting the managemental behavior of the chosen resources, the Birch Murnaghan equation of state [23] was employed.

For the computation of volume optimization, aiming to identify the least stable unit cell, we minimized the energy of the unit cell. Utilizing Birch Murnaghan’s equation of state, the values are methodically determined to guess the ground state distinctiveness of the unit cell. The individuality includes the bulk modulus (B), its pressure derivative (B′), and the equilibrium lattice constant (a_o_). The minimum energy (E_o_) corresponding to the lowest volume value (V_o_) represents the actual lowest state of the complexes. Composites having extra negative energy are deemed to have a more stable structure. The improved managing parameters, such as a_o_, E_o_, V_o_, B_o_, and B′, are presented in Table 1.

Notably, the computed results reveal that the bulk modulus increases as the lattice constantly rises, aligning with the general trend expected from this methodology. This consistency underscores the precision and accuracy of the computed results.

**Figure 2:**
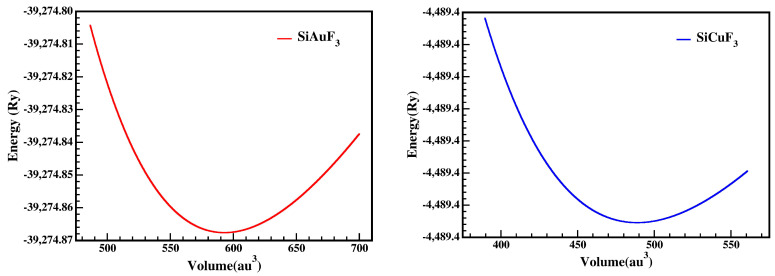
A representation of SiJF_3_’s (M = Au, Cu) optimized crystal curve from Birch Murnaghan’s equation.

By looking into the optimization graphs, we can see that the crystal SiCuF_3_ has more negative energy as shown in Figure 2; hence, one can claim that the crystal SiCuF_3_ is more structurally stable than the crystal SiAuF_3_. For a comparison of the structures, the reader is referred to Table 1.

### 2.2. Electronic Properties

**Figure 3:**
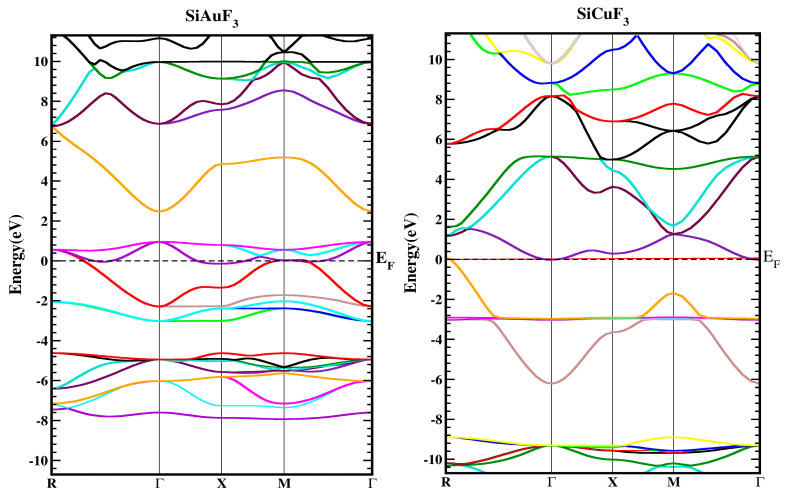
Represent the band structure of SiJF_3_ (J = Au and Cu) crystals using the TB-mBJ approach.

In Figure 3, the band structures of SiJF_3_ (J = Au and Cu) are depicted utilizing the TB-mBJ approximation. It is essential to emphasize that results employing Local Density Approximation (LDA) and Generalized Gradient Approximation (GGA) notably diminish the critical band gap of semiconductors and dielectrics [22,23]. This reduction arises due to the inconsistency in reconstructing the exchange–correlation energy and its charge derivative. To address this issue, the “The modified Becke Johnson” potential (TB-MBJ) has been employed successfully in various contemporary studies [14,24,25].

For the symmetric geometry of SiJF_3_ (J = Au and Cu), the energy band structures in the Brillouin zones have a higher symmetric direction, as illustrated in Figure 3. The Fermi energy level, denoted by E_F_, is represented by a horizontal line that separates the lower band from the upper band. The region above this level is termed the upper band, while the region below the Fermi level is referred to as the lower band, as illustrated in the diagram below.

**Figure 4:**
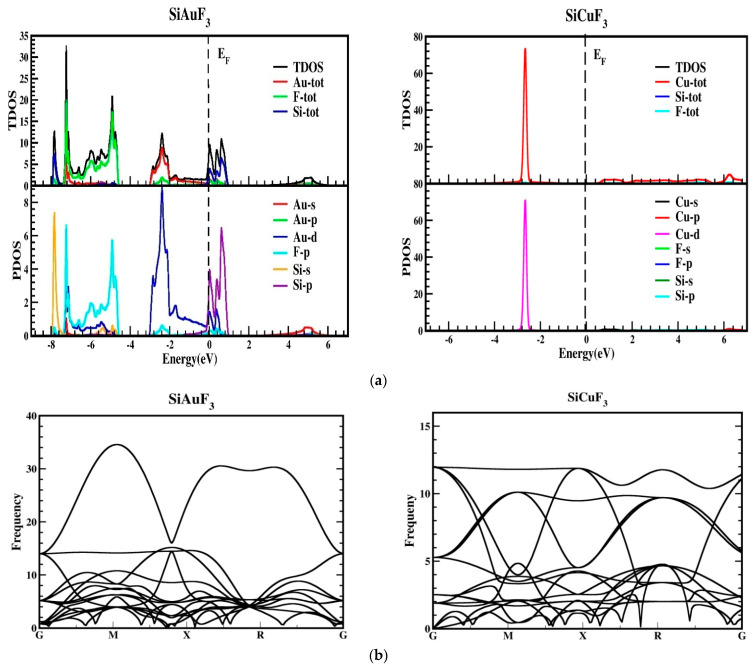
(**a**) Total density of states (TDOSs) and partial density of states (PDOS) of SiJF_3_ (J = Au and Cu) crystals using the TB-mBJ approach. (**b**) A representation of the phonopy band structure of SiJF_3_ (M = Au and Cu).

As depicted in Figure 3, the metallic attributes of SiAuF_3_ and SiCuF_3_ manifest due to the intersection of the valence band peak and the conduction band valley with the Fermi line. To gain a more comprehensive insight into the electronic states of SiJF_3_ (where J can be either Au or Cu), the ensuing illustration showcases both the overall density and the computed partial densities of the states. Each band is symbolized by a vertical marker representing the Fermi level, where the left side corresponds to the lower band and the right side is allocated for the upper band. The specific roles of diverse electronic states within the valence and conduction bands are elucidated by distinctive lines annotated in each graph presented in Figure 4. The energy range for the DOS of SiAuF_3_ is from −8.0 to 6.1 eV whereas that of SiCuF_3_ rages from −0.31 to 0.86 eV. Significant contributions to different states in the valance and conduction band are explained as follows. First, we present the details of SiAuF_3_. In the valance band, Au-tot, Au-d, F-tot, F-p, Si-tot, and Si-p are the major contributors. The Au-tot contribution ranges from −8.6 to 0 eV, with the highest peak of 10 corresponding to −2.3 eV. The Au-d contribution ranges from −8.1 to 0 eV, with the highest peak of 10 at −2.4 eV. The F-tot contribution ranges from −8.0 to 0 eV, with the highest peak of 6 at −4.8 eV. The F-p contribution ranges from −8.6 to 0 eV, with the highest peak of 6 at −4.9 eV. The Si-tot contribution ranges from −8.0 to 0 eV, with the highest peak of 6 at −4.8 eV. Similarly, the Si-p contribution ranges from −8.1 to 0 eV, with the highest peak of 6 at −4.8 eV. The influences stemming from all other states, such as Au-s, Au-p, and Si-p, are negligible. The description of the density of states (DOSs) for the SiCuF_3_ crystal is elucidated as follows. In the valence band, contributions of Cu-tot and Cu-d are maximum. The range of contribution of Cu-tot is from −6.9 to 0 eV, with the highest peak of 99 at −2.9 eV. The contribution of Cu-d is from −6.9 to 0 eV, with the highest peak of 100 at −2.9. The contributions from the other states are very small. Similarly, in the conduction band, the contributions of all states are very small (up to 2).

### 2.3. Elastic Properties

The response of a particular compound to externally applied forces is utilized to determine the flexible properties of the material. The information found from these calculations provides insights into the stability and toughness of the crystal. These material properties were computed under no pressure by identifying the stress tensor’s parts for slight distortions and adding up energy in accordance with lattice deformation while maintaining volume integrity [26]. The IRelast program, integrated with the Wien2k software (version 18.2), was employed to determine elastic constants, utilizing all relevant details of the cubic system. Key elastic constants, namely *C*_11_*, C*_12_, and *C*_44_, which offer detailed information about any cubic crystal system, were calculated and are presented in Table 2. Certain rules must be satisfied for a cubic crystal to be mechanically stable, including *C*_11_ being greater than *C*_12_, *C*_11_ being greater than 0, *C*_44_ being greater than zero, *C*_11_ + 2 *C*_12_ *> 0*, and the bulk modulus B being greater than 0 [27].

Our calculated elastic constants meet all the aforementioned conditions, leading to the conclusion that our compounds are mechanically stable. According to our computed data, the *C*_11_ value for SiAuF_3_ is 79.10 GPa, and for SiCuF_3_, it is 90.50 GPa. This indicates that the crystal SiCuF_3_ is somewhat stiffer than the crystal SiAuF_3_.

The thermodynamic stability was also confirmed by performing the phonon calculation, as revealed in Figure 4b, and it demonstrates that all reported frequencies for both the compounds SiAuF_3_ and SiCuF_3_ are real and that there is no imaginary frequency [28,29]. This suggests that both of the chemicals listed have thermodynamic stability.

The anisotropic constant, denoted as “*A*”, provides insights into a crystal’s ability to generate minute cracks. Engineers typically employ these facts to analyze the tiny response of a complex to exterior stress. The “*A*” value for our chosen amalgam is designed based on the aforementioned elastic constants, utilizing the formula mentioned below [30].

(1) $$A=\frac{2\times C_{44}}{C_{11}-C_{12}}$$

For a material to exhibit to be isotropic, the value of “*A*” should be 1; any other value indicates anisotropy. The calculated “*A*” values for both of our compounds are presented in Table 2. Both compounds are found to be anisotropic since “*A*” is not equal to 1, and the degree of variation determines the extent of anisotropy. As shown in Table 2, the calculated “*A*” value for SiAuF_3_ is 0.35, while that for SiCuF_3_ is 0.055, indicating that SiCuF_3_ is more anisotropic than SiAuF_3_. Additional essential factors such as Young’s modulus (*E*), shear modulus (*G*), and Poisson’s ratio (ν) are computed using elastic constants and are listed in Table 2. The formulas used to derive these parameters are provided below [31]:

(2) $$E=\frac{9\times B\times G}{G+3\times B}$$

(3) $$v=\frac{3\times B-2\times G}{2\left(G+2\times B\right)}$$

(4) $$G_{v}=\frac{C_{11}-C_{12}+3\times C_{44}}{5}$$

(5) $$G_{R}=\frac{5\times C_{44}\left(C_{11}-C_{12}\right)}{4\times C_{44}+3C_{11}-C_{12}}$$

Important properties include brittleness and ductility to study the structure of any particular crystal. Cauchy’s pressure, which is given as (*C*_11_*–C*_44_), is the parameter that can tell us about a crystal’s ductility [32]. It works in the following way: the material will be ductile if there is a positive difference between these constants; otherwise, it will be brittle. Both of our crystals have positive Cauchy’s pressures, 75.42 GPa for SiAuF_3_ and 89.14 GPa for SiCuF_3_, demonstrating that they both possess the ductile quality. Keeping in view of the above calculated data, it can be claimed that our selected crystals, SiJF_3_ (J = Au and Cu), are robust, fracture resistant, anisotropic, and mechanically ductile. Materials with such characteristics have vast applications in the field of modern-day technology.

### 2.4. Optical Properties

To determine the optical characteristics of the chosen crystals, we employed incident photons within the energy spectrum of 0 to 14 eV. The calculated equilibrium lattice constant and the dielectric function ε(ω) were utilized to derive all optical properties for both substances.

#### 2.4.1. The Dielectric Function

**Figure 5:**
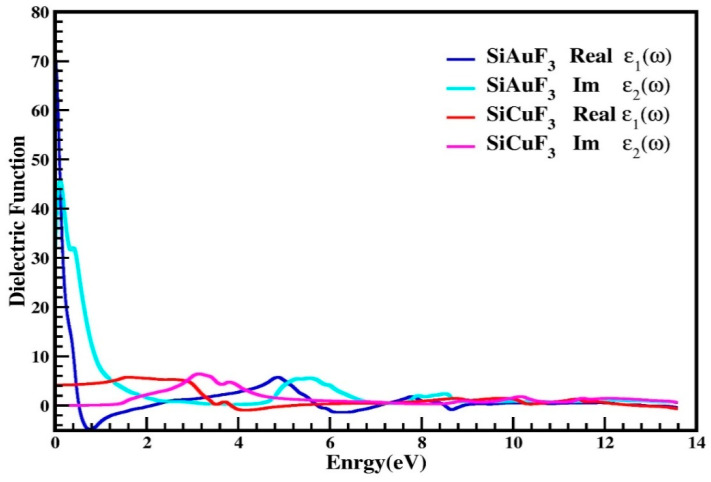
Dielectric function *ε*(ω) of SiJF_3_ molecules (J = Au and Cu).

The dielectric function is denoted by “*ε*(*ω*)” and is described by equation *ε*(*ω*) = *ε*_1_(*ω*) + *iε*_2_(*ω*). The farmer part is real while the latter is imaginary. Figure 5 explains the distribution of incoming photons by the mentioned compounds and renders electrical polarizability. It is clear from the graph of Figure 5, for the crystal SiAuF_3,_ that the dielectric function value is 70 at 0 eV, which is the maximum value of ε_1_(ω), and this value for SiCuF_3_ at 0.0 eV is 2. According to the Penn model [33,34], the larger ε_1_(0) value leads to lesser band gaps and vice versa. In Figure 5, ε_2_(ω) values are displayed for the energy up to 14.0 eV. Using the ε_2_(ω) spectrum, we discovered that the first critical maxima for AuSiF_3_ and SiCuF_3_ occur at 0.10 eV and 3.10 eV, respectively. At the X-symmetries point, a direct optical transition occurs from the lower to the higher band, initiated by the absorption edge. The curve initiates an ascent and descent immediately upon surpassing the energy barrier. In the energy range between 9.0 eV and 13.6 eV, both the real and imaginary components of the dielectric permittivity demonstrate congruent behavior.

#### 2.4.2. Index of Refraction

**Figure 6:**
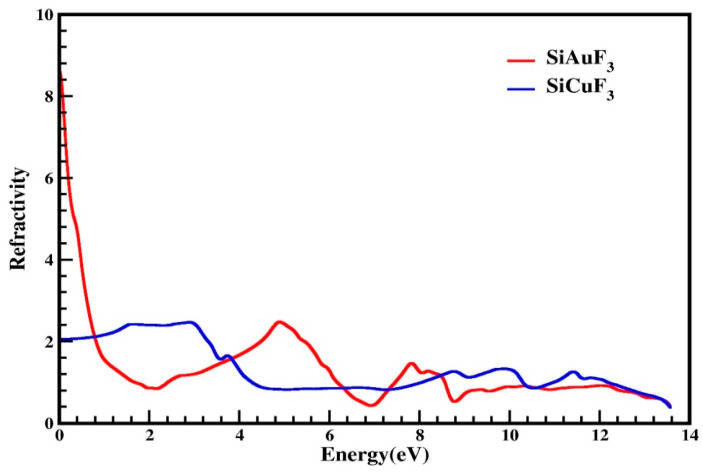
Calculated refractive index of SiJF_3_ compounds (J = Au and Cu), which diminishes at higher energies. Noteworthy peaks in the refractive index for SiAuF_3_ are observed at 8.6, 2.5, 1.5, and 0.6, corresponding to 0.0, 5.0, 7.8, and 12.2 eV, respectively.

Utilizing the computed values of ε_1_(ω) and ε_2_(ω), various parameters can be extrapolated for the computation of diverse optical properties in any crystal. These parameters include conductivity “σ(ω)”, the absorption coefficient “I(ω)”, the refractive index “η(ω)”, and reflectivity “R(ω)”. Figure 6 is devoted to representing the refractive index values that were determined computationally for the SiJF_3_ crystals (J = Au and Cu). As is clear from the graph shown in Figure 6, this value at 0.0 eV, which is denoted by η(_0_), for SiAuF_3_ is 8.6 and that of the crystal SiCuF_3_ is 2.0. This graph also highlights a substantial gap in the refractive indices η(ω) for both compounds at 0 eV.

The refractive index peaks of SiCuF_3_ are 2.2, 2.3, 1.7, 1.3, 1.4, and 1.2 at 1.6, 3.0, 3.8, 8.8, 9.9, and 11.4 eV, respectively. By analyzing the computed data, we can say that the refractive index of SiCuF_3_ is greater than 1 for a photon energy from zero to 4.3 eV. then it is falls and its value becomes less than 1 till 7.4. For an energy of greater than 7.4 eV, again this value becomes greater than 1 up to 12.0 eV. Substances with a higher quantity of electrons typically exhibit greater refractivity. Consequently, any procedure that augments electron density results in an increase in the refractive index of the material.

#### 2.4.3. The Absorption Coefficient

**Figure 7:**
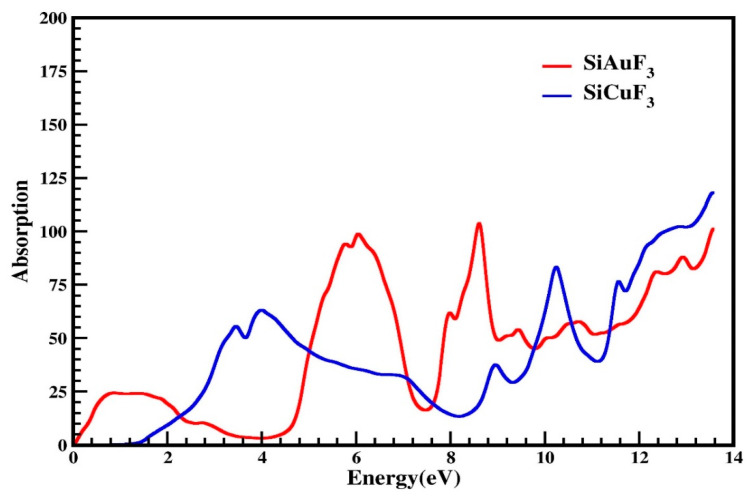
Calculated absorption coefficient of SiJF_3_ crystals (J = Au and Cu).

I(ω), the term used for the absorption coefficient, was calculated from *ε*(ω), the symbol used for the dielectric function. Our computed data are presented in Figure 7. By looking at Figure 7, it is evident that at zero electron volt, both compounds have zero absorption coefficients, and it is zero till 0.1 eV for SiAuF_3_ and till 1.2 eV for SiCuF_3_ crystal. This means that the threshold energy for SiCuF_3_ is 1.2 eV and that of SiAuF_3_ is 0.1 eV. After this, the absorption of both the compounds increases and reaches 25 at 0.8 eV for SiAuF_3_ and 54 at 3.5 eV for SiCuF_3_. The absorption value for SiAuF_3_ once again decreases and reaches 2.5 at 3.8 till 4.2 eV. The fluctuated values of I(ω) for SiAuF_3_ are 25, 95, 100, 65, 105, 55, 60, 80, 90, and 105 at 0.8, 5.8, 6.2, 8.0, 8.6, 9.4, 10.8, 12.3, 12.9, and 13.6 eV, respectively. For the crystal SiCuF_3_, some mountains in the graph of Figure 7 include 56, 65, 40, 85, 77, 105, and 120 which are observed at 3.2, 4.0, 8.9, 10.2, 11.3, and 13.5 eV, respectively. From the above discussion, we can say that energy gaps where SiAuF_3_ is a good absorber are from 0.1 eV to 2.2 eV, from 5.0 eV to 7.0 eV, from 7.6 eV to 9.7 eV, and from 10.6 eV to 11.4 eV. Similarly, the SiCuF_3_ analogues are from 2.3 eV to 4.9 eV, from 7.1 eV to 7.5 eV, from 9.8 eV to 10.5 eV, and from 11.5 eV to 13.6 eV.

#### 2.4.4. Reflectivity

**Figure 8:**
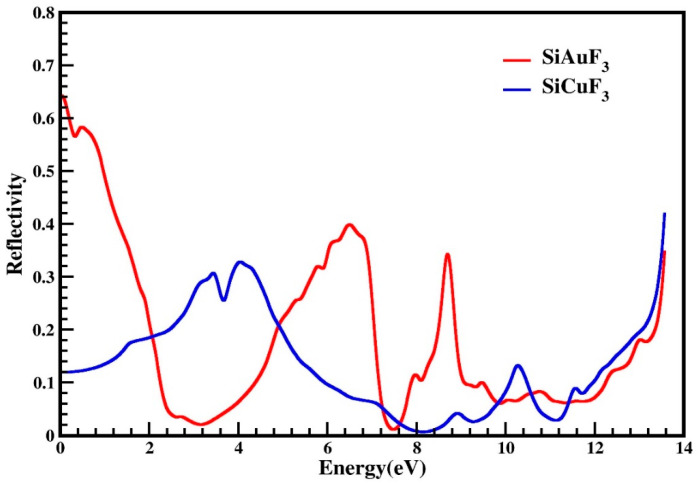
Reflectivity R(ω) of incident light from SiJF_3_ crystals (J = Au and Cu).

The reflectivity of the crystals is denoted by the symbol R(ω), and it is derived from the dielectric function. The threshold values of both the compounds are non zero and are 0.64 and the threshold values for both compounds are non zero, measuring at 0.64 for SiAuF_3_ and at 0.12 for SiCuF_3_, respectively. Some observed prominent peaks of SiAuF_3_ are 0.58, 0.4, 0.34, 0.35, and 0.35 at 0.5, 6.6, 8.7, and 13.6 eV, respectively. For the molecule SiCuF_3_, prominent peaks are at 0.31, 0.33, 0.14, and 0.42 at 3.5, 4.2, 10.3, and 13.6 eV. The zero-frequency reflectance R(0) for SiAuF_3_ and SiCuF_3_ is 0.64 and 0.12, respectively. We know that the more the reflectivity of the crystal, the less the transparency is. If we compare the reflectivity of both the compounds, we can note that the energy ranges where SiAuF_3_ is more transparent: from 2.1 eV to 4.8 eV, from 7.3 eV to 7.6 eV, from 9.9 eV to 10.5 eV, and from 11.3 eV to 13.6 eV. Likewise, intervals of energy where SiCuF3 exhibits heightened transparency include from 0.0 eV to 2.0 eV, from 4.9 eV to 7.2 eV, from 7.7 eV to 9.8 eV, and from 10.6 eV to 11.2 eV. A comprehensive illustration of this scenario is presented in Figure 8. Materials characterized by excellent transparency are advisable for crafting efficient lens materials.

#### 2.4.5. Optical Conductivity

**Figure 9:**
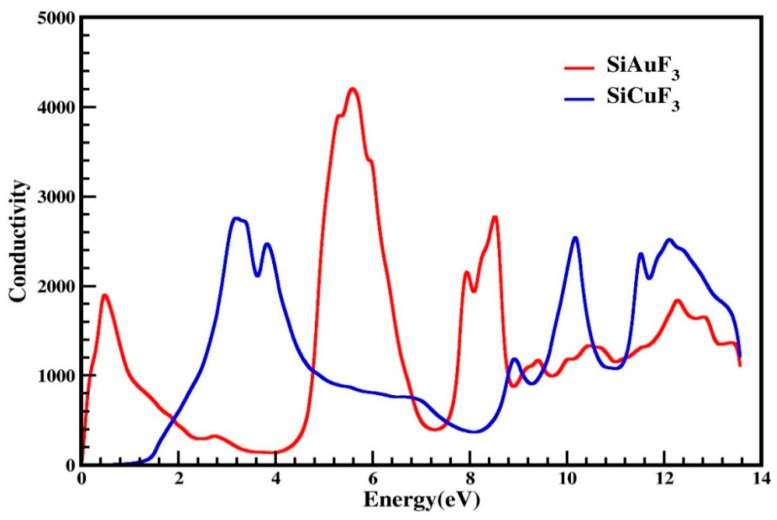
Optical conduction σ(ω) through SiJF_3_ (J = Au and Cu) compounds.

The symbol σ(ω) is employed to denote optical conductivity and is defined as, at a specific frequency for any given material, establishing the relationship between the induced current density and the absolute value of the induced electric field within the substance. This attribute serves to illustrate the conduction of photons within a material. Utilizing the dielectric function, we have illustrated photon conductivity in Figure 9. As is evident from this graph, for both of our designated crystals, photon conductivity is absent at 0 eV and remains zero up to 0.1 eV for SiAuF_3_ and persists at zero up to 0.8 eV for SiCuF_3_. After this, its value for SiAuF_3_ increases abruptly to 1900 at 0.6 eV and to the highest value of 4200 at 5.6 eV, whereas for SiCuF_3_, it starts increasing and reaches 2800 at 3.2 eV, which is the maximum peak value for SiCuF_3._ For SiCuF_3_, the value of conductivity reaches 2800, 2500, 1200, 2600, 2400, and 2600 at 3.2, 3.9, 8.9, 10.1, 11.4, and 12.0 eV, respectively. Similarly, the same graph for SiAuF_3_, shown in Figure 9, gives different peaks of 1900, 4200, 2100, 2750, 1200, 1300, and 1800 at 0.5, 5.6, 7.8, 8.4, 9.2, 10.4, and 12.2, respectively. From the above discussion, one can say that the energy ranges where SiAuF_3_ is more conductive are from 0.1 to 1.8 eV, from 4.8 to 6.8, from 7.5 to 8.6, and from 9.0 to 9.4. Similarly, the SiCuF_3_ analogues are from 1.9 to 4.7, from 6.9 to 7.4, from 8.7 to 8.9, and from 9.5 to 13.6 eV.

#### 2.4.6. The Energy Loss Function (ELF)

**Figure 10:**
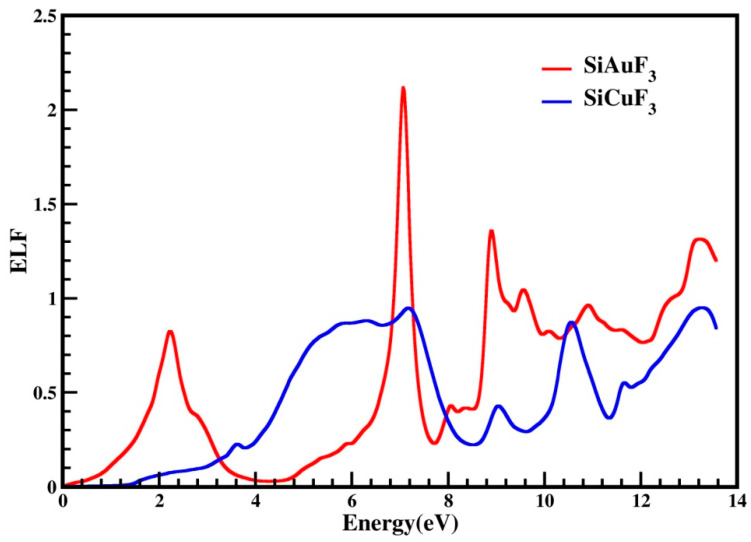
An illustration of the representation of the optical energy loss function, denoted as L(ω), for SiJF_3_ (J = Au and Cu).

ELF is the function that tells us about decreases in the energy of an electron during its motion in a particular compound. It may be helpful in defining the interdependency of inter-bands, intra-bands, and Plasmons. In Figure 10, the computed energy loss incurred by an electron is presented for both compounds. The above-mentioned figure indicates that the ELF value for CuSiF_3_, our selected compound, is zero from 0 to 1.4 eV, and for SiAuF_3_, it is from 0 to 0.2 eV. For SiAuF_3_, the ELF value after 0.2 eV continuously increases and decreases, having a maximum peak of 2.1 at 6.9 eV. For SiCuF_3_, the ELF value after 1.4 eV continuously increases and decreases, having the highest peak of 0.9 at 7.2 eV. So, we can say that at a low energy of up to 0.2 eV, AuSiF_3_ does not absorb any electron energy. Similarly, CuSiF_3_ does not absorb any electron energy up to 1.4 eV. From 0.2 to 3.2 eV, SiAuF_3_ is a better absorber than SiCuF_3_. After 3.2 eV, SiCuF_3_ takes its turn and becomes a better absorber than SiAuF_3_ up to 6.8 eV. From 6.8 to 7.1 eV, SiAuF_3_ takes its turn once again. From 7.1 to 7.8 eV, SiCuF_3_ once again becomes more absorbent than SiAuF_3._ After 7.8 eV, SiAuF_3_ again takes its turn and becomes an excellent absorber for electron energy. From the graph, it is clear that SiAuF_3_ absorbs the maximum energy at 6.9 eV while SiCuF_3_ does the same at 7.2 eV.

## 3. Computational Methodology

The Full Potential Linear Augmented Plane Wave (FP-LAPW) approach, implemented through the WIEN2k software, was employed for the computation of the aforementioned properties, as discussed in [35]. Our choice of the TB-mBJ method stems from its utility in determining the density of states (DOSs) and various optical and electronic properties [36]. In parallel, the Generalized Gradient Approximation (GGA) and the exchange–correlation potential were instrumental in calculating the structural and elastic characteristics, serving as valuable tools for such computations [37].

For the FP-LAPW basis functions considered in our study, a muffin-tin sphere radius (RMT) of 8, with the smallest radius, was selected. The maximum k-point value, denoted as Kmax, was determined within our model’s plane wave expansion to achieve a significant level of convergence. In the case of the J = (Au and Cu), F, and Si crystals, the RMT values for the muffin-tin spheres were set at 2.13, 2.0, and 2.5 atomic units (au). Within the muffin-tin spheres, the spherical harmonics were extended to Imax = 11, while the charge density was reduced to Gmax = 12 (au) using a Fourier expansion. Convergence in the self-consistent field calculations was deemed achieved when the net energy reduction fell within the 0.001 Ry energy range.

To determine the equation of state, we applied the Birch–Murnaghan approach by comparing the energy–volume curve [38,39]. Elastic constants were derived using the IRelast program to understand structural behaviors [26]. For extracting the optical properties of our chosen crystals, we relied on the dielectric function ε(ω) [40,41].

## 4. Conclusions

In this study, we conducted a comprehensive comparative analysis of the structural, electronic, optical, and elastic properties of SiAuF_3_ and SiCuF_3_ halide perovskites using computational methods. Our findings provide valuable insights into the characteristics and potential applications of these materials.

Structural Properties:

- Both SiAuF_3_ and SiCuF_3_ crystallize in a cubic structure with the Pm-3m space group.
- SiCuF_3_ is structurally more stable than SiAuF_3_, as indicated by its lower energy and higher bulk modulus.

Electronic Properties:

- Both compounds exhibit metallic behavior, with the valence and conduction bands touching the Fermi energy level.
- The band structures and density of states (DOSs) were analyzed, revealing the contributions of various electronic states in the valence and conduction bands.

Elastic Properties:

- Both SiAuF_3_ and SiCuF_3_ are mechanically stable, meeting the criteria for cubic crystal stability.
- SiCuF_3_ is more anisotropic than SiAuF_3_, with a lower anisotropic constant (A).

Optical Properties:

- SiCuF_3_ has a larger refractive index at lower energies, indicating greater transparency in that range.
- SiAuF_3_ has higher reflectivity at certain energy ranges, making it less transparent in those regions.

Both compounds exhibit distinct absorption coefficient peaks, revealing their ability to absorb light at specific energy levels.

The energy loss function (ELF) analysis shows that SiAuF_3_ and SiCuF_3_ have different absorption behaviors at different energy ranges, with SiAuF_3_ absorbing the maximum energy at 6.9 eV and SiCuF_3_ at 7.2 eV.

In summary, SiAuF_3_ and SiCuF_3_ exhibit different optical and mechanical properties, with SiCuF_3_ generally showing more favorable characteristics for transparency and mechanical stability. These findings provide valuable information for researchers exploring the potential applications of these halide perovskite materials in various fields, including optoelectronics and energy storage. Further research and experimentation are warranted to harness the unique properties of these compounds for practical applications.

## Figures and Tables

**Table 1:** Data computed from optimized crystal unit cells of BJO_3_ (J = Cr and Mn) compounds.

Crystals	a_o_ (Å)	B (GPa)	B′	V_0_ (a.u^3^)	E_0_ (Ry)
**SiAuF_3_**	4.45	64.98	5.22	592.81	−39,274.87
**SiCuF_3_**	4.17	59.02	5.13	488.70	−4489.42

**Table 2:** SiJF_3_ (J = Au and Cu) molecules.

Compounds	SiAuF_3_	SiCuF_3_
***C*_11_**	79.10	90.50
***C*_12_**	57.95	41.08
***C*_44_**	3.68	1.36
***G***	5.71	6.44
***A***	0.35	0.055
***v***	0.45	0.42
***B*/*G***	11.38	9.16

## Data Availability

Data are contained within the article.
